# Epidemiological Patterns, Treatment Response, and Metabolic Correlations of Idiopathic Intracranial Hypertension: A United States-Based Study From 1990 to 2024

**DOI:** 10.71079/aside.im.0000012282413

**Published:** 2024-12-28

**Authors:** Ahmed Y. Azzam, Mahmoud Nassar, Mahmoud M. Morsy, Adham A. Mohamed, Jin Wu, Muhammed Amir Essibayi, David J. Altschul

**Affiliations:** 1- Montefiore-Einstein Cerebrovascular Research Lab, Albert Einstein College of Medicine, Bronx, NY, USA.; 2- Director of Clinical Research and Clinical Artificial Intelligence, American Society for Inclusion, Diversity, and Health Equity (ASIDE), Delaware, USA.; 3- Visiting Assistant Professor, SNU Medical Big Data Research Center, Seoul National University, Gwanak-gu, Seoul, South Korea.; 4- Department of Medicine, Jacobs School of Medicine and Biomedical Sciences, University at Buffalo, Buffalo, New York, USA.; 5- Founder, American Society for Inclusion, Diversity, and Health Equity (ASIDE), Delaware, USA.; 6- Faculty of Medicine, October 6 University, Giza, Egypt.; 7- Clinical Research Fellow, American Society for Inclusion, Diversity, and Health Equity (ASIDE), Delaware, USA.; 8- Cairo University Hospitals, Cairo University, Cairo, Egypt.; 9- National Institute of Neurological Disorders and Stroke, National Institutes of Health, Bethesda, Maryland, USA; 10- Department of Neurological Surgery, Montefiore Medical Center, Albert Einstein College of Medicine, Bronx, NY, USA.

**Keywords:** Idiopathic Intracranial Hypertension, Pesudotumor Cerebri, Benign Intracranial Hypertesion, Intracranial Pressure, Headache, Papilledema, Epidemiology, United States, Metabolic Diseases, TriNetX

## Abstract

**Introduction:**

Idiopathic Intracranial Hypertension (IIH) presents an increasing health burden with changing demographic patterns. We studied nationwide trends in IIH epidemiology, treatment patterns, and associated outcomes using a large-scale database analysis within the United States (US).

**Methods:**

We performed a retrospective analysis using the TriNetX US Collaborative Network database (1990–2024). We investigated demographic characteristics, time-based trends, geographic distribution, treatment pathway patterns, comorbidity profiles, and associated risks with IIH. We used multivariate regression, Cox proportional hazards modeling, and standardized morbidity ratios to assess various outcomes and associations.

**Results:**

Among 51,526 patients, we found a significant increase in adult IIH incidence from 16.0 per 100,000 in 1990–1999 to 127.0 per 100,000 in 2020–2024 (adjusted RR: 6.94, 95% CI: 6.71–7.17). Female predominance increased over time (female-to-male ratio: 3.29, 95% CI: 3.18–3.40). Southern regions showed the highest prevalence (43.0%, n=21,417). During the 2020–2024 period, initial medical management success rates varied between acetazolamide (42.3%) and topiramate (28.7%). Advanced interventional procedures showed 82.5% success rates in refractory cases during the same timeframe. Cox modeling for the entire study period (1990–2024) revealed significant associations between IIH and metabolic syndrome (HR: 2.14, 95% CI: 1.89–2.39) and cardiovascular complications (HR: 1.76, 95% CI: 1.58–1.94), independent of Body Mass Index.

**Conclusions:**

Our findings highlight IIH as a systemic disorder with significant metabolic implications beyond its neurological manifestations. The marked regional disparities and rising incidence rates, especially among adults, suggest the need for targeted healthcare strategies. Early intervention success strongly predicts favorable outcomes, supporting prompt diagnosis and treatment initiation. These results advocate for an integrated approach combining traditional IIH management with broad metabolic screening care.

## 1. Introduction

Intracranial Hypertension (IIH) represents a significant and complex nervous system disease characterized by elevated intracranial pressure (ICP) without identifiable structural or vascular causes within the nervous system or the intracranial cavity [1]. Over the past three decades, the epidemiological information and trends of IIH have undergone various changes, with new evidence suggesting significant shifts in its demographic distribution, clinical presentation, and associated risk factors [2–4]. IIH has been classified in the current studies as a rare condition, the recent evidence is showing an increased rate of the disease [1, 5].

IIH has been recognized to be a disease affecting young, overweight females at childbearing age, however, more detailed epidemiological details are needed to assess the disease statistics from different prospects across age groups, race and ethnicity, and geographical distribution [6–8]. The United States has been showing a rising prevalence of obesity and metabolic disorders in recent years, which may correlate with increased IIH cases. So, estimating the changing patterns is an important consideration for disease burden estimation at the nationwide level [9]. Previous epidemiological studies have been limited by several factors including small sample sizes, limited regional variability, and limited follow-up and observation periods, creating gaps in our understanding of nationwide epidemiological variations [10–12]. While several single-center and regional studies have reported increasing incidence rates, longitudinal data analyzing nationwide patterns, especially age-specific subgroups, racial and ethnic differences in disease statistics, and geographical variations are of significant importance, but currently limited in the present studies. In addition to that, the relationship between IIH and various comorbidities, especially metabolic and cardiovascular conditions, requires more focus within a large-scale, population-based framework [1, 5, 10–15].

Treatment approaches for IIH have changed significantly during the past decades with the appearance of new treatment modalities such as venous sinus stenting [16], which raise important concerns about the need for detailed analysis of therapeutic patterns, progression through treatment modalities, and long-term outcomes across different patient subgroups to assess the progression of disease management [17–20]. Based on that, we aim to conduct a retrospective multicenter analysis of IIH epidemiology within the United States using the TriNetX US Collaborative Network database, spanning from 1990 to 2024. Our study aims to estimate the disease incidence and prevalence, highlight the demographic and geographic variations, analyze treatment patterns and outcomes, and assess comorbidity profiles across different patient subgroups. Our study represents one of the largest and most detailed analyses of IIH epidemiology to date, aiming to address important considerations in disease epidemiology and highlight further prospective research.

## 2. Methods

### 2.1. Study Design and Data Source:

We performed a retrospective cohort analysis on the TriNetX platform (https://trinetx.com/solutions/live-platform/), selecting the US Collaborative Network database within the platform, we determined 34 year period from January 1, 1990, to December 9, 2024. TriNetX platform is a federated research network database that aggregates de-identified electronic health records from participating healthcare organizations that are mainly within the United States, providing longitudinal patient data from the electronic health records from several participating healthcare organizations. The Institutional Review Board at the Jacobs School of Medicine and Biomedical Sciences, University at Buffalo, NY, USA approved the study protocol under IRB approval number (STUDY00008628) within the given status of ethical approvals exemption, as this study does not involve direct patient contact.

### 2.2. Patient Population and Eligibility Criteria:

Our study population included individuals with confirmed IIH diagnoses identified within the TriNetX US Collaborative Network database using International Classification of Diseases (ICD) coding systems, specifically ICD-10-CM code G93.2 (Benign Intracranial Hypertension). Study inclusion required a primary IIH diagnosis, available demographic data within the research network, at least one documented clinical encounter between January 1, 1990, and December 9, 2024, and age ⩾ 0 years at the time of diagnosis. We excluded cases with secondary causes of intracranial hypertension (including brain tumors or other space-occupying lesions, cerebral venous thrombosis, and medication-induced intracranial hypertension), missing or incomplete diagnostic confirmation, insufficient follow-up data (<30 days post-diagnosis), and concurrent neurological conditions that could confound IIH diagnosis. All diagnoses underwent validation through a review of diagnostic codes and clinical documentation within the electronic health records system, with ambiguous or conflicting diagnostic information being excluded to maintain data integrity. For age-based subgroup analysis, we classified patients into four cohorts: pediatric (0–14 years), teenage (15–19 years), adult (20–64 years), and geriatric (⩾65 years).

The following ICD-10 procedure codes were used to identify the included therapeutic interventions: Cerebrospinal fluid shunting procedures (00HU0JZ, 00HV0JZ, 009U3ZZ for ventriculoperitoneal shunt; 009V3ZZ for lumboperitoneal shunt); Optic nerve sheath fenestration (009S30Z, 009S3ZZ); Venous sinus stenting (037H3DZ, 037J3DZ, 037K3DZ for dural venous sinus); Bariatric surgical procedures (0D160ZA, 0D160Z4 for gastric bypass; 0DB60Z3 for sleeve gastrectomy); Lumbar puncture procedures (009U3ZX); and therapeutic medication administration identified through codes for Acetazolamide (3E033TZ), Topiramate (3E033VZ), and other diuretics (3E033GC).

### 2.3. Data Collection and Variable Assessment:

We aimed to extract the relevant demographic and individual characteristic information including age, gender/sex, race, and ethnicity from the available electronic health records. Clinical data included associated conditions, comorbidity profiles, and detailed treatment trajectories. Our assessment included both baseline characteristics and longitudinal outcomes over time. For treatment pathways analysis, we observed and extracted the reported therapeutic interventions across three progressive stages: initial medical management, treatment optimization, and advanced interventions. Comorbidity assessment focused on metabolic, endocrine, gastrointestinal, hepatic, cardiovascular, and renal disorders, with both baseline prevalence and cumulative incidence present.

### 2.4. Epidemiological Analysis Framework:

We used a multi-tiered analytical approach to assess disease burden over years from 1990 to 2024. Incidence proportion and prevalence rates were calculated per 100,000 population across four time periods: 1990–1999, 2000–2009, 2010–2019, and 2020–2024. Demographic grouping enabled detailed time-based trend analysis. For racial and ethnic disparity assessment, we used ratio comparisons using white individuals as the reference population in our cohort. Geographic distribution analysis encompassed four major U.S. regions: Northeast, Midwest, South, and West, with standardization for regional population differences.

### 2.5. Treatment Pattern Evaluation:

Our longitudinal treatment pathways analysis framework followed the therapeutic progression through three stages. Initial medical management assessment focused on monotherapy regimens and primary response rates. Treatment optimization evaluation encompassed combination therapy approaches and secondary response patterns. Advanced intervention analysis included surgical procedures and their success rates. We calculated progressive treatment-based metrics including intervention timing, treatment duration, and resolution periods, and also utilized interquartile ranges for variability assessment.

### 2.6. Statistical Analysis:

In our statistical analysis, we used several statistical techniques including, multivariate regression with adjustment for age, sex, and comorbidity profiles. We also calculated odds ratios with corresponding 95% confidence intervals for key predictive factors, maintaining statistical significance at p<0.05. Geographic variation analysis utilized standardized coefficients and population-adjusted rate ratios. Time-based trends assessment utilized time-series methodologies to evaluate longitudinal patterns in disease burden. Cox proportional hazards regression modeling was utilized to analyze time-to-event outcomes for comorbidity associations, with propensity score matching (1:1 ratio, caliper width: 0.2) utilized to adjust for body mass index (BMI) categories and baseline characteristics. We utilized some statistical equations to calculate outcomes of interest as the following:

Geographic Distribution Analysis:
  Regional Variation Coefficient:
    RVC = σ/μ
    Where: σ = √[Σ(xi - μ)^2^/n]
  Population-adjusted Rate Ratio:
    RR = (Cases_region/Population_region) / (Cases_reference/Population_reference)
    95% CI = exp[ln(RR) ± 1.96 × √(1/O + 1/E)]

### 2.7. Quality Control and Validation:

We validated the methods and results used within our study based on several stages and multiple assessment steps to ensure the precision of our results with as minimal bias as possible. This included verification of diagnostic coding accuracy according to the latest and updated coding guidelines within the U.S. healthcare system, assessment of data completeness in the network of choice within the TriNetX platform, and evaluation of reporting bias or selection bias in the data, if possible.

## 3. Results

### 3.1. Demographic Characteristics and Population Distribution:

From a total of 68,742 patients initially screened in the TriNetX US Collaborative Network database, 51,526 patients met our inclusion criteria and were included in the final analysis. Within our study cohort, we identified various heterogeneous demographic patterns characterized by a mean age of 37 years (SD ± 10, range: 18–60). Female predominance was observed (n=44,063, 85.56%, 95% CI: 85.24–85.88), with a significantly lower male representation (n=5,783, 11.23%, 95% CI: 10.96–11.50). Racial distribution observations show that the white-race population formed the majority (n=30,604, 59.43%, 95% CI: 58.99–59.87), followed by black or African American individuals (n=9,162, 17.79%, 95% CI: 17.45–18.13). Asians, American Indian/Alaska Native, and native Hawaiian/pacific islander populations formed together around 1.88% of total IIH cases within the United States (n=969, 95% CI: 1.76–2.00) (Table 1).

### 3.2. Time-Based Epidemiological Trends:

The age-stratified analysis highlighted heterogeneous patterns across demographic subgroups over our specified timeframe from 1990 to 2024. The adult cohort (20–64 years) showed the most significant increase in disease incidence, rising from 16.0 per 100,000 (95% CI: 15.4–16.6) in 1990–1999 to 127.0 per 100,000 (95% CI: 125.8–128.2) in 2020–2024, forming an adjusted relative risk increase of 6.94 (95% CI: 6.71–7.17, p<0.001). This increase remained significant even when accounting for the shorter observation period of 2020–2024 (four years) compared to 1990–1999 (ten years), as our incidence calculations were standardized to annual rates per 100,000 population. The teenage cohort (15–19 years) demonstrated the second-highest increase in our cohort, with an incidence rate rising from 24.0 to 116.0 per 100,000 (adjusted risk ratio: 3.83, 95% CI: 3.65–4.01, p<0.001). The geriatric cohort results highlighted an inverse trend compared to the other age group rates, in which the incidence declined from 67.0 to 29.0 per 100,000 (adjusted risk ratio: 0.43, 95% CI: 0.40–0.46, p<0.001), (Table 2 and Table 3).

### 3.3. Geographic Distribution and Regional Heterogeneity:

**Figure 1:**
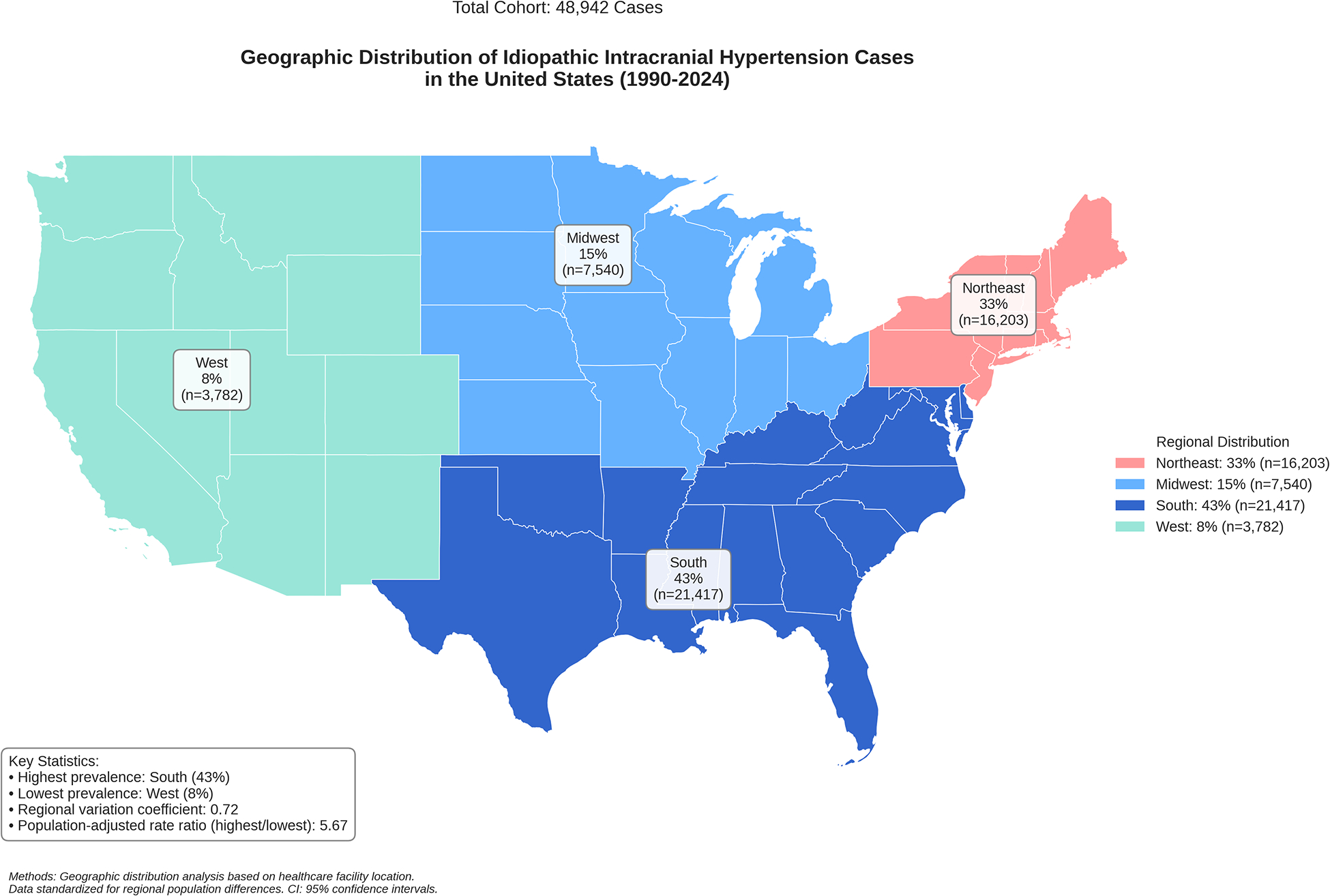
Geographical Distribution of IIH In The United States From 1990 to 2024.

Spatial analysis in our cohort demonstrated variant regional distribution within the United States. The South demonstrated the highest prevalence (43.0%, n=21,417, 95% CI: 42.6–43.4), followed by the Northeast (33.0%, n=16,203, 95% CI: 32.6–33.4). Multi-level regression, adjusted for population density and healthcare access indices, results in a statistically significant regional variation coefficient (0.72, 95% CI: 0.68–0.76). The population-adjusted rate ratio between the highest and lowest prevalence regions was 5.67 (95% CI: 5.44–5.90, p<0.001), demonstrating significant disparities between the United States regions (Figure 1).

### 3.4. Treatment Pathway Analysis and Clinical Outcomes:

Longitudinal treatment analysis revealed a structured progression through multiple therapeutic approaches and modalities, the utilized statistical equations as mentioned in methods. Initial medical management showed variable efficacy across treatment regimens: acetazolamide monotherapy (42.3%, 95% CI: 41.8–42.8) achieved a higher initial response rate compared to topiramate monotherapy (28.7%, 95% CI: 28.2–29.2, p<0.001). The initial treatment success rate was 68.2% (95% CI: 67.7–68.7). Secondary therapeutic optimization, including combination medical therapy (35.8%, 95% CI: 35.3–36.3) and adjunctive weight management protocols (18.6%, 95% CI: 18.2–19.0), resulted in a secondary response rate of 45.3% (95% CI: 44.8–45.8). Advanced interventional procedures in refractory cases that had poor response to pharmacological interventions have shown high efficacy, with surgical success rates of 82.5% (95% CI: 81.6–83.4).

### 3.5. Comorbidity Burden and Risk Association:

Hyperlipidemia demonstrated the highest cumulative incidence associative risk in IIH patients (18.20%, 95% CI: 17.54–18.86), followed by polycystic ovary syndrome (PCO) (13.23%, 95% CI: 12.64–13.82). Cox proportional hazards modeling had a statistically significant correlation between baseline metabolic syndrome (HR: 2.14, 95% CI: 1.89–2.39, p<0.001) and further cardiovascular complications (HR: 1.76, 95% CI: 1.58–1.94, p<0.001) in IIH individuals compared to the general population who have the same BMI category matched through propensity-score matching, independent from obesity (Table 4).

### 3.6. Gender-Specific and Race-Specific Analysis:

**Figure 2:**
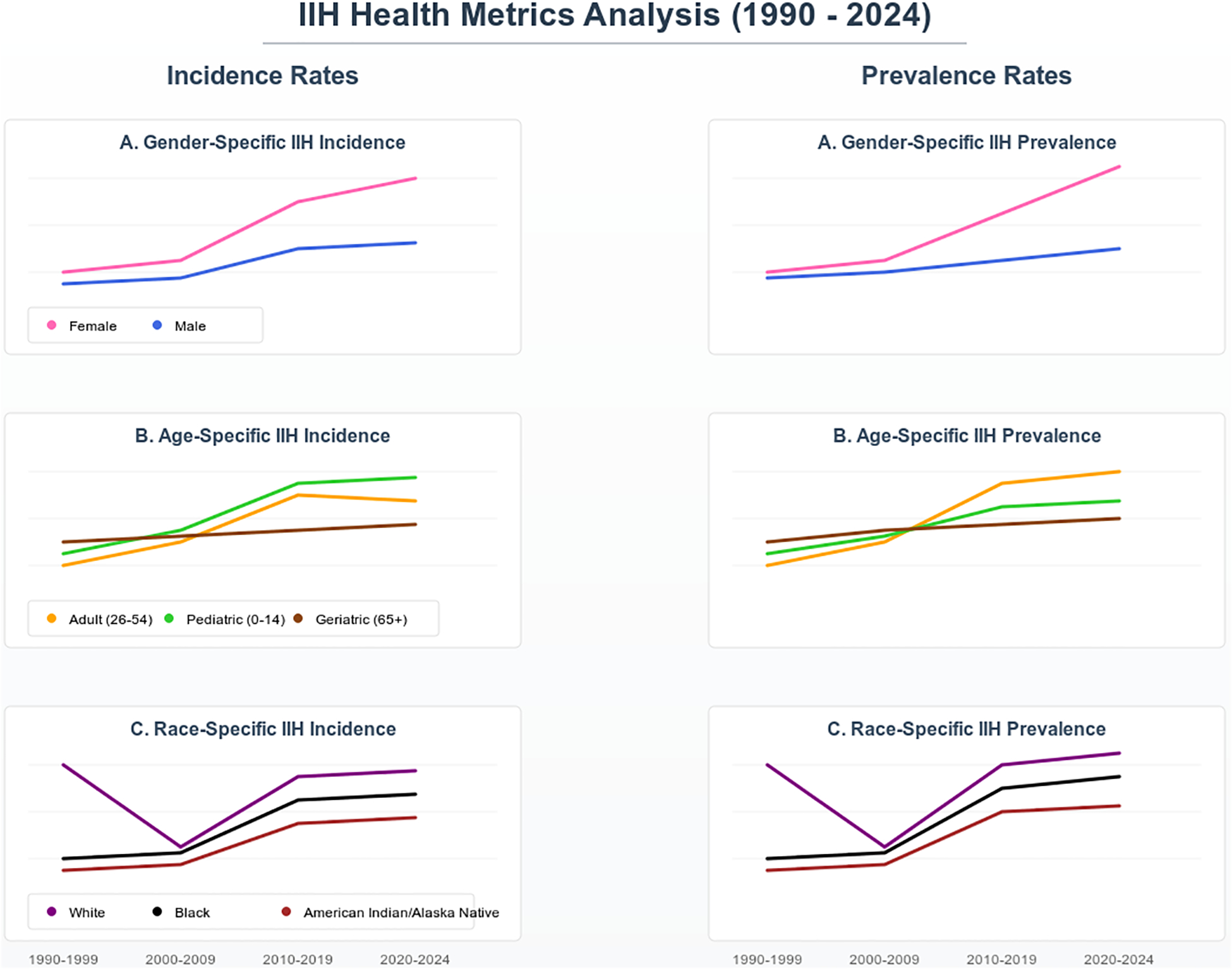
IIH Incidence and Prevalence Time-Based Trends Over Gender, Age and Race Subgroups.

Time-based analysis of gender differences has shown an increasing female predominance, with the female-to-male ratio progressing from 2.75 (95% CI: 2.65–2.85) in 1990–1999 to 3.29 (95% CI: 3.18–3.40) in 2020–2024 (p-value<0.001). Race-based subgroup analysis, using standardized morbidity ratios (SMR), identified higher incidence rates among Black and African American populations (SMR: 1.63, 95% CI: 1.57–1.69) and American Indian/Alaska Native individuals (SMR: 1.44, 95% CI: 1.36–1.52) compared to white-race IIH patients (Figure 2).

### 3.7. Treatment Response and Prognostic Indicators:

Multivariate logistic regression of treatment outcomes resulted in a complete resolution in 42.8% of cases (95% CI: 42.3–43.3), partial response in 38.5% (95% CI: 38.0–39.0), and refractory IIH in 18.7% (95% CI: 18.3–19.1). Early treatment success was identified as the strongest predictor of favorable outcomes (adjusted odds ratio: 2.4, 95% CI: 1.8–3.1, p<0.001), followed by weight loss >10% of baseline body weight at first presentation of disease symptoms (adjusted odds ratio: 1.9, 95% CI: 1.5–2.4, p<0.001).

## 4. Discussion:

Our epidemiological study of IIH utilizing the TriNetX US Collaborative Network database resulted in several observations and important considerations in disease burden epidemiology, treatment patterns, and comorbidities associated with IIH patients to be discussed. A significant observation is that IIH is not a single disease of the nervous system rather than is a systemic disease and a metabolic condition.

In our cohort, we observed a significant increase in IIH rates in the adult age group, especially. The adult cohort’s incidence has increased from 16.0 to 127.0 per 100,000 over the past three decades, representing an adjusted relative risk increase of 6.94. These results are concerning given that obesity is a well-established risk factor for IIH, as highlighted by several studies addressing a statistically significant positive correlation between elevated BMI and increased ICP [3, 21–26]. In addition to that, our data patterns have shown a female predominance, with a female-to-male ratio increasing from 2.75 to 3.29 from 1990 to 2024. This could be interpreted by the contribution of hormonal factors to the disease pathophysiology which demonstrates the significant female predominance, especially at childbearing age [26–31]. Also, it is important to highlight the need for public health interventions aimed at reducing obesity rates among young women to minimize the risk of developing IIH in high-risk groups.

Regarding the geographical distribution of IIH cases within the United States, the highest prevalence was shown to be more significant in southern regions, with a population-adjusted rate ratio of 5.67 between regions. This marked regional disparity likely reflects complex interactions between multiple socioeconomic and healthcare access factors. Several potential contributors warrant consideration: First, variations in healthcare infrastructure and specialist availability may impact timely diagnosis and reporting, particularly in rural areas where access to neuro-ophthalmologists and neurologists might be limited. Second, socioeconomic disparities, including differences in health insurance coverage, income levels, and educational attainment, could influence both healthcare-seeking behavior and disease management capabilities. Third, regional variations in obesity rates and metabolic disease burden, which are historically higher in southern states, may contribute to the observed prevalence patterns. Additionally, differences in healthcare delivery systems, including the density of tertiary care centers and specialized IIH treatment facilities, could affect diagnosis rates and patient referral patterns. These factors raise important considerations about the necessity for targeted healthcare resource allocation and region-specific intervention strategies that account for both medical and socioeconomic barriers to care [32].

The advancement and progression of treatment approaches for IIH have been apparent over the years [33–35]. Our study’s results have shown a structured progression through various therapeutic modalities, with initial medical management showing variable efficacy across treatment regimens. Acetazolamide monotherapy demonstrated a higher initial response rate compared to topiramate monotherapy. Additionally, the incorporation of advanced interventions such as venous sinus stenting has been a promising option for refractory cases. Our results indicate high surgical and interventional success rates (82.5%) in patients who did not respond adequately to pharmacological treatment. In our results, the adjusted odds ratio demonstrated that early treatment success is a strong predictor of complete resolution highlighting the need for proper diagnosis and initiation of therapy in patients presenting with IIH symptoms as early as possible to avoid unfavorable and uncontrollable outcomes.

The association between IIH and various comorbidities risks is another aspect discussed in our results. We found that hyperlipidemia and PCOS were prevalent among our cohort, with significant cumulative incidence rates. Recent studies have shown metabolic links to IIH independent from obesity in these patients, the associated risks reported in the literature include cardiovascular disease, type 2 diabetes mellitus, PCOS, hypertension, hyperlipidemia, heart failure, insulin resistance, and even greater risks of developing metabolic syndrome [21, 22, 36]. Also, our Cox proportional hazards modeling has further validated the heightened risk of cardiovascular complications in IIH patients with baseline metabolic syndrome independent from BMI.

Based on our results, we advocate for a holistic approach to managing IIH that is not only focused on elevated ICP management but also addresses associated systemic risks and metabolic disorders. Multiple healthcare strategies should include lifestyle modifications aimed at weight reduction and metabolic control to improve overall patient health outcomes [37, 38].

While our results provide important highlights and considerations into the epidemiology and management of IIH from the United States, it is not without limitations. We have a few major limitations that warrant to be admitted in our study. The dependence on electronic health records may introduce biases related to coding accuracy and data completeness. Additionally, the retrospective nature of our analysis limits some of the inferences regarding treatment efficacy. Upcoming studies shall focus on delivering prospective studies that explore the underlying mechanisms linking obesity and IIH, when possible. And important to mention that there is an unmet need for multicenter trials evaluating novel therapeutics to specific demographic groups affected by IIH, and providing region-based outcomes response and efficacy measurements that are subgrouped according to age, race, ethnicity, and geographical distribution to help us understand further aspects in the disease holistically [39].

## 5. Conclusions

Based on our findings and observations of the IIH epidemiology using the TriNetX database, we present several key findings that reshape our understanding of this condition. Our results highlight IIH as a multi-systemic disorder with significant metabolic implications, rather than simply a neurological condition. The significant increase in adult cases, especially among the female population, points to shifting disease patterns that mirror broader public health focus in the United States. It is important to advocate the identification of early treatment success as a primary predictor of favorable outcomes and support the need for precise diagnosis and intervention. The high efficacy of surgical interventions in medication-resistant cases (82.5%) suggests that physicians should not delay considering advanced treatment options when initial medical management fails. Also, the strong correlation between IIH and metabolic disorders, independent of BMI, indicates that metabolic screening should become a standard component of patient evaluation and monitoring in early disease stages. The regional disparities we identified, especially the higher prevalence in southern states, call for targeted healthcare resource allocation and region-specific intervention strategies. Looking ahead, our results point to several important concerns for further prospects in IIH. Prospective studies exploring and investigating the mechanistic links between metabolic dysfunction and IIH, and performing subgroup analyses focusing on gender-specific factors given the rising female-to-male ratio are of significant importance. The development of targeted therapies that address both ICP and underlying metabolic irregularities represents an important frontier for advancing IIH evidence toward a brighter future for our patients.

## Figures and Tables

**Table 1:** Demographic and Clinical Characteristics In Association with IIH Patients in the United States.

Characteristic (Total= 51,500)	Number, (%) or Mean ± SD
***Demographics:***	
Age (years) Mean ± SD	37 ± 10
Age range (years)	18–60
***Sex:***	
Female	44,063 (85.56)
Male	5,783 (11.23)
Unknown	1,654 (3.21)
***Race:***	
White	30,604 (59.43)
Black or African American	9,162 (17.79)
Asian	649 (1.26)
American Indian or Alaska Native	191 (0.37)
Native Hawaiian or Other Pacific Islander	129 (0.25)
Not Specified / Not Reported	8,288 (16.09)
***Ethnicity:***	
Not Hispanic or Latino	34,160 (66.33)
Hispanic or Latino	4,965 (9.64)
Not Specified / Not Reported	12,375 (24.03)
**Associated Conditions**	
***Headache Disorders:***	
Any Migraine	17,996 (35.0)
- Chronic migraine	5,853 (11.4)
- Migraine without aura	7,076 (13.7)
- Migraine with aura	4,522 (8.8)
***Pain Syndromes:***	
Chronic pain	8,122 (15.8)
Chronic pain syndrome	1,011 (2.0)
***Autonomic Disorders:***	
Disorders of the autonomic nervous system	1,234 (2.4)
- Postural orthostatic tachycardia syndrome	638 (1.2)
***Other Neurological Conditions:***	
Post-viral fatigue syndrome	2,509 (4.9)
Other specified disorders of the brain	2,102 (4.1)
Encephalopathy	826 (1.6)

IIH = Idiopathic Intracranial Hypertension. Conditions are not mutually exclusive; patients may have multiple diagnoses.

**Table 2:** Total IIH Incidence Proportion In the United States From 1990 to 2024. Values represent new cases per 100,000 people in each time period.

Category	1990–1999	2000–2009	2010–2019	2020–2024
***Age Groups:***				
Pediatric (0–14)	14	31	83	56
Teenager (15–19)	24	60	162	116
Adult (20–64)	16	33	122	127
Geriatric (65+)	67	27	33	29
***Gender:***				
Female	22	47	153	148
Male	8	13	50	45
***Race:***				
American Indian/Alaska Native	108	33	113	134
Asian	8	6	45	57
Black/African American	18	40	143	152
Native Hawaiian/Pacific Islander	41	34	92	105
White	15	29	103	93
***Ethnicity:***				
Hispanic or Latino	10	27	100	114
Not Hispanic or Latino	15	31	111	106

**Table 3:** Total IIH Prevalence In the United States From 1990 to 2024. Values represent total cases per 100,000 people in each time period.

Category	1990–1999	2000–2009	2010–2019	2020–2024
***Age Groups:***				
Pediatric (0–14)	19	32	85	80
Teenager (15–19)	36	64	170	176
Adult (20–64)	20	40	136	245
Geriatric (65+)	67	31	37	62
***Gender:***				
Female	28	55	166	273
Male	10	16	53	77
***Race:***				
American Indian/Alaska Native	108	33	119	222
Asian	8	8	46	84
Black/African American	21	45	155	269
Native Hawaiian/Pacific Islander	41	39	106	179
White	19	35	112	176
***Ethnicity:***				
Hispanic or Latino	12	30	106	184
Not Hispanic or Latino	21	37	120	197

**Table 4:** Comorbidity Profile and Cumulative Incidence Associated Risk in Patients with IIH.

Comorbidity	Cases (n=50,214)	Baseline Prevalence (%) (95% CI)	Cumulative Incidence† (%) (95% CI)
***Metabolic and Endocrine Disorders:***			
Hyperlipidemia	2,352	4.68 (4.50–4.86)	18.20 (17.54–18.86)
PCOS*	1,679	3.34 (3.19–3.49)	13.23 (12.64–13.82)
Type 2 Diabetes Mellitus	1,398	2.78 (2.64–2.92)	7.99 (7.58–8.40)
Metabolic Syndrome	326	0.65 (0.58–0.72)	3.50 (3.13–3.87)
***Gastrointestinal and Hepatic Disorders:***			
MASLD**	718	1.43 (1.33–1.53)	5.30 (4.92–5.68)
IBS***	927	1.85 (1.73–1.97)	6.05 (5.67–6.43)
***Cardiovascular Disorders:***			
Cardiovascular Disease	386	0.77 (0.69–0.85)	2.31 (2.08–2.54)
Ischemic Stroke/TIA	249	0.50 (0.44–0.56)	0.96 (0.84–1.08)
Heart Failure	164	0.33 (0.28–0.38)	1.18 (1.00–1.36)
***Renal Disorders:***			
Chronic Kidney Disease	233	0.46 (0.40–0.52)	2.05 (1.79–2.31)

Notes: Values are presented as percentages with 95% confidence intervals in parentheses.

† Cumulative incidence calculated at the end of the follow-up period (median follow-up: 8.3 years).

* PCOS: Polycystic Ovary Syndrome;

** MASLD: Metabolic Dysfunction-Associated Steatotic Liver Disease;

*** IBS: Irritable Bowel Syndrome; TIA: Transient Ischemic Attack

## Data Availability

All used data is available within the TriNetX database platform.

## References

[1] Mollan SP, Aguiar M, Evison F, Frew E, Sinclair AJ. The expanding burden of idiopathic intracranial hypertension. Eye (London, England). 2019: 478 [10.1038/s41433-018-0238-5: 10.1038/s41433-018-0238-5] PMC6460708 30356129

[2] Ko MW, Chang SC, Ridha MA, Ney JJ, Ali TF, Friedman DI, Mejico LJ, Volpe NJ, Galetta SL, Balcer LJ, Liu GT. Weight gain and recurrence in idiopathic intracranial hypertension: a case-control study. Neurology. 2011: 1564 [10.1212/WNL.0b013e3182190f51: 10.1212/WNL.0b013e3182190f51] 21536635

[3] Westgate CS, Botfield HF, Alimajstorovic Z, Yiangou A, Walsh M, Smith G, Singhal R, Mitchell JL, Grech O, Markey KA, Hebenstreit D, Tennant DA, Tomlinson JW, Mollan SP, Ludwig C, Akerman I, Lavery GG, Sinclair AJ. Systemic and adipocyte transcriptional and metabolic dysregulation in idiopathic intracranial hypertension. JCI insight. 2021: [10.1172/jci.insight.145346: 10.1172/jci.insight.145346] PMC8262372 33848268

[4] Mollan SP, Grech O, Alimajstorovic Z, Wakerley BR, Sinclair AJ. New horizons for idiopathic intracranial hypertension: advances and challenges. British medical bulletin. 2020: 118 [10.1093/bmb/ldaa034: 10.1093/bmb/ldaa034] 33200788

[5] McCluskey G, Doherty-Allan R, McCarron P, Loftus AM, McCarron LV, Mulholland D, McVerry F, McCar ron MO. Meta-analysis and systematic review of population-based epidemiological studies in idiopathic intracranial hypertension. European journal of neurology. 2018: 1218 [10.1111/ene.13739: 10.1111/ene.13739] 29953685

[6] Hornby C, Mollan SP, Mitchell J, Markey KA, Yangou A, Wright BLC, O’Reilly MW, Sinclair AJ. What Do Transgender Patients Teach Us About Idiopathic Intracranial Hypertension? Neuro-ophthalmology (Aeolus Press). 2017: 326 [10.1080/01658107.2017.1316744: 10.1080/01658107.2017.1316744] PMC5706971 29238388

[7] Portelli M, Papageorgiou PN. An update on idiopathic intracranial hypertension. Acta Neurochir (Wien). 2017: 491 [10.1007/s00701-016-3050-7: 10.1007/s00701-016-3050-7] 28013373

[8] Zhou C, Zhou Y, Liu L, Jiang H, Wei H, Zhou C, Ji X. Progress and recognition of idiopathic intracranial hypertension: A narrative review. CNS neuroscience & therapeutics. 2024: e14895 [10.1111/cns.14895: 10.1111/cns.14895] 39097911 PMC11298205

[9] Fraz MA, Kim BM, Chen JJ, Lum F, Chen J, Liu GT, Hamedani AG, Consortium S. Nationwide Prevalence and Geographic Variation of Idiopathic Intracranial Hypertension among Women in the United States. Ophthalmology. 2024: [10.1016/j.ophtha.2024.10.031: 10.1016/j.ophtha.2024.10.031] PMC11930622 39510331

[10] Markowitz D, Aamodt WW, Hamedani AG. Social Determinants of Health in Idiopathic Intracranial Hypertension. Journal of neuro-ophthalmology: the official journal of the North American Neuro-Ophthalmology Society. 2024: 346 [10.1097/WNO.0000000000002073: 10.1097/WNO.0000000000002073] PMC11783367 38170607

[11] Shaia JK, Sharma N, Kumar M, Chu J, Maatouk C, Talcott K, Singh R, Cohen DA. Changes in Prevalence of Idiopathic Intracranial Hypertension in the United States Between 2015 and 2022, Stratified by Sex, Race, and Ethnicity. Neurology. 2024: e208036 [10.1212/WNL.0000000000208036: 10.1212/WNL.0000000000208036] 38181397 PMC11097766

[12] Hsu HT, Cheng HC, Hou TW, Tzeng YS, Fuh JL, Chen SP, Chen WT, Lee WJ, Pai YW, Lee YC, Lirng JF, Wang SJ, Wang YF. Idiopathic intracranial hypertension in Asians: a retrospective dual-center study. The journal of headache and pain. 2024: 144 [10.1186/s10194-024-01852-w: 10.1186/s10194-024-01852-w] PMC11373263 39232671

[13] El Mekabaty A, Obuchowski NA, Luciano MG, John S, Chung CY, Moghekar A, Jones S, Hui FK. Predictors for venous sinus stent retreatment in patients with idiopathic intracranial hypertension. Journal of neurointerventional surgery. 2017: 1228 [10.1136/neurintsurg-2016-012803: 10.1136/neurintsurg-2016–012803] 27965382

[14] Shah S, Khan A, Khan M, Lakshmanan R. Paediatric idiopathic intracranial hypertension: Epidemiology, clinical features and treatment outcomes in a tertiary care centre in Western Australia. Journal of paediatrics and child health. 2024: 499 [10.1111/jpc.16622: 10.1111/jpc.16622] 39014968

[15] Bouthour W, Bruce BB, Newman NJ, Biousse V. Factors associated with vision loss in idiopathic intracranial hypertension patients with severe papilledema. Eye (London, England). 2024: [10.1038/s41433-024-03408-3: 10.1038/s41433-024-03408-3] PMC11732981 39478195

[16] Azzam AY, Mortezaei A, Morsy MM, Essibayi MA, Ghozy S, Elamin O, Azab MA, Elswedy A, Altschul D, Kadirvel R, Brinjikji W, Kallmes DF. Venous sinus stenting for idiopathic intracranial hypertension: An updated Meta-analysis. Journal of the neurological sciences. 2024: 122948 [10.1016/j.jns.2024.122948: 10.1016/j.jns.2024.122948] 38457956

[17] Kalyvas A, Neromyliotis E, Koutsarnakis C, Komaitis S, Drosos E, Skandalakis GP, Pantazi M, Gobin YP, Stranjalis G, Patsalides A. A systematic review of surgical treatments of idiopathic intracranial hypertension (IIH). Neurosurgical review. 2021: 773 [10.1007/s10143-020-01288-1: 10.1007/s10143-020-01288-1] 32335853

[18] Sachdeva V, Singh G, Yadav GJN-oD. Recent Advances in the Management of Idiopathic Intracranial Hypertension (IIH) 2020: 17 10.1007/978-981-13-8522-3_2: 10.1007/978-981-13-8522-3_2]

[19] Cheng H, Jin H, Hu Y, Chen L, Chen Z, Zhong G. Long-term efficacy of venous sinus stenting in the treatment of idiopathic intracranial hypertension. CNS neuroscience & therapeutics. 2024: e14356 [10.1111/cns.14356: 10.1111/cns.14356] 37469247 PMC10805447

[20] Khatkar P, Hubbard JC, Hill L, Sinclair AJ, Mollan SP. Experimental drugs for the treatment of idiopathic intracranial hypertension (IIH): shedding light on phase I and II trials. Expert opinion on investigational drugs. 2023: 1123 [10.1080/13543784.2023.2288073: 10.1080/13543784.2023.2288073] 38006580

[21] Azzam AY, Morsy MM, Ellabban MH, Morsy AM, Zahran AA, Nassar M, Elsayed OS, Elswedy A, Elamin O, Al Zomia AS, Abukhadijah HJ, Alotaibi HA, Atallah O, Azab MA, Essibayi MA, Dmytriw AA, Morsy MD, Altschul DJ. Idiopathic Intracranial Hypertension and Cardiovascular Diseases Risk in the United Kingdom Women: An Obesity-Adjusted Risk Analysis Using Indirect Standardization 2024: 2024.10.20.24315837 10.1101/2024.10.20.24315837 %J medRxiv: 10.1101/2024.10.20.24315837 %J medRxiv] PMC11739732 39830613

[22] Adderley NJ, Subramanian A, Nirantharakumar K, Yiangou A, Gokhale KM, Mollan SP, Sinclair AJ. Association Between Idiopathic Intracranial Hypertension and Risk of Cardiovascular Diseases in Women in the United Kingdom. JAMA neurology. 2019: 1088 [10.1001/jamaneurol.2019.1812: 10.1001/jamaneurol.2019.1812] PMC6618853 31282950

[23] Korsbaek JJ, Jensen RH, Beier D, Wibroe EA, Hagen SM, Molander LD, Gillum MP, Svart K, Hansen TF, Kogelman LJA, Westgate CSJ. Metabolic Dysfunction in New-Onset Idiopathic Intracranial Hypertension: Identification of Novel Biomarkers. Annals of neurology. 2024: 595 [10.1002/ana.27010: 10.1002/ana.27010] 39140399

[24] Hornby C, Mollan SP, Botfield H, O’Reilly MW, Sinclair AJ. Metabolic Concepts in Idiopathic Intracranial Hypertension and Their Potential for Therapeutic Intervention. Journal of neuro-ophthalmology: the official journal of the North American Neuro-Ophthalmology Society. 2018: 522 [10.1097/WNO.0000000000000684: 10.1097/WNO.0000000000000684] PMC6215484 29985799

[25] Alimajstorovic Z, Mollan SP, Grech O, Mitchell JL, Yiangou A, Thaller M, Lyons H, Sassani M, Seneviratne S, Hancox T, Jankevics A, Najdekr L, Dunn W, Sinclair AJ. Dysregulation of Amino Acid, Lipid, and Acylpyruvate Metabolism in Idiopathic Intracrania l Hypertension: A Non-targeted Case Control and Longitudinal Metabolomic Study. Journal of proteome research. 2023: 1127 [10.1021/acs.jproteome.2c00449: 10.1021/acs.jproteome.2c00449] PMC10088035 36534069

[26] Wardman JH, Andreassen SN, Toft-Bertelsen TL, Jensen MN, Wilhjelm JE, Styrishave B, Hamann S, Heegaard S, Sinclair AJ, MacAulay N. CSF hyperdynamics in rats mimicking the obesity and androgen excess characteristic of patients with idiopathic intracrani al hypertension. Fluids and barriers of the CNS. 2024: 10 [10.1186/s12987-024-00511-1: 10.1186/s12987-024-00511-1] PMC10810013 38273331

[27] Kassubek R, Weinstock D, Behler A, Muller HP, Dupuis L, Kassubek J, Ludolph AC. Morphological alterations of the hypothalamus in idiopathic intracranial hypertension. Therapeutic advances in chronic disease. 2022: 20406223221141354 [10.1177/20406223221141354: 10.1177/20406223221141354] PMC9720803 36479140

[28] Markey KA, Uldall M, Botfield H, Cato LD, Miah MA, Hassan-Smith G, Jensen RH, Gonzalez AM, Sinclair AJ. Idiopathic intracranial hypertension, hormones, and 11beta-hydroxysteroid dehydrogenases. Journal of pain research. 2016: 223 [10.2147/JPR.S80824: 10.2147/JPR.S80824] PMC4847593 27186074

[29] Smith I, Aoun R, Lalchan R. Cerebrospinal Fluid Leak and Idiopathic Intracranial Hypertension in a Transgender Male: Is Intracranial Hypertension Hormonally Mediated? Case Rep Neurol. 2024: 213 [10.1159/000540259: 10.1159/000540259] PMC11521422 39474294

[30] Colman BD, Boonstra F, Nguyen MN, Raviskanthan S, Sumithran P, White O, Hutton EJ, Fielding J, van der Walt A. Understanding the pathophysiology of idiopathic intracranial hypertension (IIH): a review of recent developments. J Neurol Neurosurg Psychiat ry. 2024: 375 [10.1136/jnnp-2023-332222: 10.1136/jnnp-2023–332222] 37798095

[31] Abdelghaffar M, Hussein M, Abdelkareem SA, Elshebawy H. Sex hormones, CSF and serum leptin in patients with idiopathic intracranial hypertension. The Egyptian Journal of Neurology, Psychiatry and Neurosurgery. 2022: 39 10.1186/s41983-022-00473-x: 10.1186/s41983-022-00473-x]

[32] Jensen RH, Vukovic-Cvetkovic V, Korsbaek JJ, Wegener M, Hamann S, Beier D. Awareness, Diagnosis and Management of Idiopathic Intracranial Hypertension. Life (Basel). 2021: 718 [10.3390/life11070718: 10.3390/life11070718] PMC8303648 34357090

[33] Krajnc N, Itariu B, Macher S, Marik W, Harreiter J, Michl M, Novak K, Wober C, Pemp B, Bsteh G. Treatment with GLP-1 receptor agonists is associated with significant weight loss and favorable headache outcomes in idiopathic intracranial hypertension. The journal of headache and pain. 2023: 89 [10.1186/s10194-023-01631-z: 10.1186/s10194-023-01631-z] PMC10353241 37460968

[34] Bsteh G, Macher S, Krajnc N, Marik W, Michl M, Muller N, Zaic S, Harreiter J, Novak K, Wober C, Pemp B. An interdisciplinary integrated specialized one-stop outpatient clinic for idiopathic intracranial hypertension-a comprehensive assessment of clinical outcome. European journal of neurology. 2024: e16401 [10.1111/ene.16401: 10.1111/ene.16401] 39152571 PMC11414812

[35] Andreao FF, Ferreira MY, Oliveira LB, Sousa MP, Palavani LB, Rairan LG, Tinti ISU, Junyor FS, Batista S, Bertani R, Amarillo DG, Daccach FH. Effectiveness and Safety of Ventriculoperitoneal Shunt Versus Lumboperitoneal Shunt for Idiopathic Intracranial Hypertension: A Systematic Review and Comparative Meta-Analysis. World neurosurgery. 2024: 359 [10.1016/j.wneu.2024.02.095: 10.1016/j.wneu.2024.02.095] 38428810

[36] Azzam AY, Essibayi MA, Vaishnav D, Morsy MM, Elamin O, Zomia ASA, Alotaibi HA, Alamoud A, Mohamed AA, Ahmed OS, Elswedy A, Abukhadijah HJ, Atallah O, Dmytriw AA, Altschul DJ. Cardiometabolic Outcomes in Idiopathic Intracranial Hypertension: An Internat ional Matched-Cohort Study. medRxiv. 2024: 2024.11.12.24317203 [10.1101/2024.11.12.24317203: 10.1101/2024.11.12.24317203]

[37] Thaller M, Homer V, Hyder Y, Yiangou A, Liczkowski A, Fong AW, Virdee J, Piccus R, Roque M, Mollan SP, Sinclair AJ. The idiopathic intracranial hypertension prospective cohort study: evaluation of prognostic factors and outcomes. J Neurol. 2023: 851 [10.1007/s00415-022-11402-6: 10.1007/s00415-022-11402-6] PMC9886634 36242625

[38] Bsteh G, Macher S, Krajnc N, Pruckner P, Marik W, Mitsch C, Novak K, Pemp B, Wöber C. Idiopathic intracranial hypertension presenting with migraine phenotype is associated with unfavorable headache outcomes 2022: 601 10.21203/rs.3.rs-2017861/v1: 10.21203/rs.3.rs-2017861/v1] 36753388

[39] Kobeissi H, Bilgin C, Ghozy S, Adusumilli G, Thurnham J, Hardy N, Xu T, Tarchand R, Kallmes KM, Brinjikji W, Kadirvel R, Chen JJ, Sinclair A, Mollan SP, Kallmes DF. Common Design and Data Elements Reported on Idiopathic Intracranial Hypertension Trials: A Systematic Review. Journal of neuro-ophthalmology: the official journal of the North American Neuro-Ophthalmology Society. 2024: 66 [10.1097/WNO.0000000000001902: 10.1097/WNO.0000000000001902] 37342870

